# Assessment of afoxolaner efficacy against *Otodectes cynotis* infestations of dogs

**DOI:** 10.1186/s13071-016-1924-4

**Published:** 2016-12-09

**Authors:** Doug Carithers, Jordan Crawford, Christa de Vos, Alta Lotriet, Josephus Fourie

**Affiliations:** 1 Merial, Inc, 3239 Satellite Blvd, Duluth, 30096 GA USA; 2 Clinvet International (Pty) Ltd, PO Box 111869321, Universitas, South Africa

**Keywords:** *Otodectes cynotis*, Dog, Afoxolaner, NexGard

## Abstract

**Background:**

The efficacy of a single 2.5 mg/kg dose of afoxolaner (NexGard®, Merial) against induced *Otodectes cynotis* infestations was assessed in eight afoxolaner-treated dogs, compared to eight untreated dogs.

**Methods:**

After *O. cynotis* infestations were established and confirmed by otoscopic assessments in 16 dogs, all of the dogs were included in the study and allocated to two separate treatment groups. The first group of eight ear mite-infested dogs remained untreated, while afoxolaner was administered orally to the second group of dogs at the minimum recommended dose once on Day 0. Otoscopic assessments performed on all dogs (Days -7, -2, 14 and 28) confirmed the presence or absence of live mites throughout the study. No serious adverse events were recorded throughout the study, and no adverse events were likely related to the administration of NexGard.

**Results:**

By Day 28, seven out of eight untreated dogs were still infested with ear mites, while only two out of eight afoxolaner-treated dogs were infested, with one and four ear mites, respectively. On Day 28, the reductions of mite counts in the afoxolaner-treated group *versus* those of the control dogs were 98.5% based on geometric means, and 99.4% based on arithmetic means. Significantly fewer (*P* < 0.05) live mites were present in the afoxolaner-treated group than the untreated group on Day 28.

**Conclusion:**

The results of this study demonstrated that a single oral administration of afoxolaner at the minimum recommended dose is highly effective (>98%) in treating dogs with induced *O. cynotis* infestations.

## Background

The ear mite *Otodectes cynotis*, the causative organism of otodectic mange [1], is a very common non-burrowing, surface-living [2] acarian which infests cats, dogs and many other carnivores. *Otodectes cynotis* is found all over the world and primarily infests the ear canals of its host; however, it has also been found on head, back and tail in heavy infestations [1, 2]. The irritation caused by the mites often results in the host scratching its ears and shaking its head, which can lead to lacerations associated with the affected ear(s) or the formation of an auricular hematoma. Secondary yeast and bacterial infections often occur in the presence of heavy ear mite infestations.

Transmission of ear mites occurs by direct contact with an infested host [1–3], most commonly with the transmission of the mites from mother to kittens or puppies before weaning. Clinical signs which can lead to the suspicion of otodectic mange include bilaterally pruritic otitis with dry, blackish-brown cerumen in the host’s ear canal; self-induced ear lesions; aural hematomas; and pruritic lesions on the face and neck. Diagnosis is usually made through otoscopic examination of the ear and microscopic examination of cerumen, which usually contains ear mites of all life stages (eggs, larvae, nymphs and adults). Additionally, skin scrapings can occasionally reveal ear mites [3]. It is important to note that, if at least one pet in the household is infested with ear mites, all pets in the household need to be treated for ear mites, as the mites are highly contagious [1, 4].

Since *O. cynotis* is a very common parasite of dogs and cats, it is essential to find effective treatments for infested pets. Historically, treatment necessitated ear flushing and cleansing with a mild ceruminolytic agent. Typical treatments include mineral oil or acaricides (monosulfiram, permethrin, or amitraz [3, 4]) directly administered into the ear canal.

Sometimes treating the ears alone would not solve the issue. Often to achieve control one would also treat the whole body with an acaricide dip or topical treatment, as mites can infest other parts of the body and re-infest the ears [3]. Later, systemic-topical treatments such as selamectin and imidacloprid/moxidectin spot on formulations were found to have good systemic activity against ear mites in the ear canal and on the body [2, 3, 5, 6].

Recently, a new class of insecticides/acaricides, the isoxazolines, has demonstrated excellent efficacy against *O. cynotis* and other similar parasites [7]. Afoxolaner is an isoxazoline administered monthly to protect dogs against fleas and ticks (NexGard®) [7–9]. It is administered at a minimum dose of 2.5 mg/kg, and comparative studies have shown that monthly administrations of afoxolaner provide outstanding protection against fleas and ticks [10, 11]. Afoxolaner has also demonstrated to be highly efficacious against *Sarcoptes scabiei* [12] and *Demodex canis* [13, 14]. Based on these observations, the present study was undertaken to assess the efficacy of a single oral administration of afoxolaner (NexGard) against *O. cynotis* infestations in dogs.

## Methods

### Animals

All dogs were a part of a colony at the study site, Clinvet, and were returned to the colony holding facility at the conclusion of the study. All animals were managed similarly and with due regard for their well-being. Trained personnel under the supervision of a veterinarian were responsible for the health of the dogs. All personnel involved in health assessments, data collection and physical examinations were blinded to the treatment allocation of the dogs. To prevent potential bias or unmasking, the treatment assessments were conducted in a non-systematic order. All dogs considered were over 6 months of age (age on day of treatment ranged from 2 years and ten months to 8 years and six months), and acclimatized to the study facility for at least seven days prior to being artificially infested with *O. cynotis* mites. Experimental infection was performed by harvesting mites by lavage from donor animals with patent natural infestations, and transferring approximately 50 to 100 mites, depending on intensity of infection in donor animals, into each ear of the recipient animal. From this pool of *O. cynotis* infested dogs, 16 dogs that ultimately developed established infections and also met qualification parameters as verified by a veterinarian *via* clinical examinations (Days -7 and -2) were included in the study. The study dogs weighed between 15.4 and 22.0 kg (as weighed on Day -1) (Table 1). The dogs were of mixed breed (all mongrels), and were healthy at the initiation of the study, except for clinical signs associated with the induced *O. cynotis* infestations. Nine males and seven females were included in the study, and the females were not pregnant or lactating. Once enrolled, dogs were kept individually in cages under strict isolation conditions, with no physical contact between animals possible. None of the dogs included in the study had been treated with a long-lasting topical or systemic miticidal/acaricidal/insecticidal product during the 12 weeks preceding Day 0, nor had they been treated with any isoxazolines for 6 months prior to Day 0.

**Table 1:** Individual dog information/details, as assessed on Day -1

Group 1 (negative control)			Group 2 (afoxolaner-treated)		
Animal ID	Sex	Body weight (kg)	Animal ID	Sex	Body weight (kg)
4D9 16A	M	17.8	287 466	F	15.4
4DE 28 F	F	18.4	CC3 CEE	M	19.8
86C 5B7	M	22.0	CD5 B63	F	18.4
956 36 F	F	20.2	DF4 840	M	17.2
B2C 7B5	F	18.6	DF5 29E	M	17.0
CC5 7C6	F	16.6	E15 130	M	20.8
DF5 E10	M	20.2	E17 62E	M	18.2
DF7 CD8	M	21.4	E19 161	F	19.8
Arithmetic mean body weight in kg: 19.4			Arithmetic mean body weight in kg: 18.3		

### Study design and treatment

This study was a randomized, single center, controlled efficacy study, following a randomized block design, and all evaluations of efficacy were performed by personnel in blinded conditions. Each dog was an experimental unit. For each dog, a total of > 10 live mites in at least one ear, as well as the presence of ≥ 1 live mite in the other ear, was necessary for inclusion in the study. Dogs were enrolled as they qualified, thus Day 0 was not the same calendar day for all dogs.

Once qualified, dogs were ranked within sex, in descending order of individual pre-administration (Day -2) live mite count, and were subsequently blocked into eight replicates of two dogs each. The dogs were then allocated to untreated group 1 and treated group 2. Dogs were weighed on Day -1 to ensure that, as close as possible and without under-dosing, the labeled minimum dose of afoxolaner (2.5 mg/kg) was administered using combinations of intact commercially available product (Table 2). All dogs were observed hourly for four hours post-administration, for possible adverse events from product administration.

**Table 2:** Administration of NexGard (afoxolaner) at ~2.5 mg/kg to the afoxolaner-treated group (Group 2) on Day 0

Animal ID	Body weight (kg)	Chews administered	Actual final dose (mg/kg)
2003287 466	15.4	1 × 28.3 mg & 1 × 11.3 mg	2.57
CC3 CEE	19.8	1 × 28.3 mg & 2 × 11.3 mg	2.57
CD5 B63	18.4	1 × 28.3 mg & 2 × 11.3 mg	2.77
DF4 840	17.2	4 × 11.3 mg	2.63
DF5 29E	17.0	4 × 11.3 mg	2.66
E15 130	20.8	5 × 11.3 mg	2.72
E17 62E	18.2	1 × 28.3 mg & 2 × 11.3 mg	2.80
E19 161	19.8	1 × 28.3 mg & 2 × 11.3 mg	2.57

Otoscopic assessments were performed on all dogs on Days -7, -2, 14 and 28 to confirm the initial presence, and subsequent presence or absence, of live mites.

### Ear flushing and mite counts

On Day 28, a qualitative assessment of the ear canals was performed prior to the flushing procedure to determine the presence of viable mites. This was followed by the quantitative assessment of ear mites by ear duct flushing, complete mite collection and count. The ear ducts were filled with an appropriate solution of a 5% aqueous solution of docusate sodium (3 to 4 ml per ear canal), then the ears massaged externally until sufficient melting of the ear duct content was observed. The melted solution for each ear was collected into a labeled collection container, followed by a saline flush of the ear duct into the same container. The contents of the container were drained into a 38 μm sieve, which was then rinsed with clean water immediately, and the contents for each ear were transferred separately into labeled bottles. This procedure was conducted on each ear until otoscopic examinations revealed that both ears were clean. The contents of each ear were collected, examined, and stored separately in the labeled bottles. All collected material was observed microscopically on the same day, and all observed live mites (adult and immature) were counted and recorded for each ear.

### Statistical analysis

The primary efficacy criterion was the number of live mites collected from the NexGard-treated group on Day 28, compared to that of the untreated control group on the same day. The statistical unit was the individual animal. The average percent reduction in mite counts for each group was calculated using geometric means: Efficacy (%) against ear mites = 100 × (GMC – GMT)/GMC, where GMC is the geometric mean number of live mites in dogs in the untreated group and GMT is the geometric mean number of live mites in dogs in the treated group. The groups were compared using a one-way ANOVA (Proc GLM procedure in SAS) with an administration effect after a logarithmic transformation on the mite (count + 1) data, and by using an ANOVA (Proc GLM procedure in SAS) with an administration effect on untransformed mite count data.

Additionally, due to large variability in mite counts in the negative control group, a non-parametric alternative test (the Mann-Whitney *U*-test) was used to assess efficacy by comparing median values. SAS version 9.3 TS Level 1 M2 [15] was used for all statistical analyses. Statistical significance for geometric mean and arithmetic mean assessments was declared at a two-sided *P*-value of 0.05.

## Results

### Clinical examinations and health observations

None of the clinical signs (mild pruritus, intermittent head-shaking) associated with the mite infestations observed during any of the clinical examinations were deemed as serious or affecting overall health and well-being. The examining veterinarian carefully examined and judged all enrolled dogs to be fit to continue in the study, at each assessment throughout the study. No serious health issues were recorded during the study, with only mild signs consistent with *O. cynotis* infestation observed. No adverse events related to the administration of afoxolaner were observed.

### Antiparasitic efficacy

All of the dogs were positive for the presence of ear mites prior to Day 0 of the study. For the untreated control dogs (Group 1), the number of live mites found on each dog on Day 28 ranged from 0 to 477. For the afoxolaner-treated dogs (Group 2), one dog harbored one mite, another had four live mites, with the remaining six dogs having no live mites found by Day 28. Based on geometric means, an average of 22.3 live mites per negative control dog, and an average of 0.3 live mites per afoxolaner-treated dog, was recorded after ear flushing. Calculating based on arithmetic means, an average of 109.9 live mites per negative control dog, and an average of 0.6 live mites per afoxolaner-treated dog, was recorded after ear flushing. Based on both geometric (one-way ANOVA with a treatment effect) means (ANOVA: *F*_(1,14)_ = 11.74, *P* = 0.0041) and arithmetic (Mann-Whitney *U*-test) assessments (*W*_(1)_ = 43.5, *Z* = -2.6354, *P* = 0.0084) significantly fewer live mites were present in the afoxolaner-treated group than were present in the untreated group by Day 28. The percent efficacy of NexGard (afoxolaner) against induced *Otodectes cynotis* on Day 28 was calculated to be 98.5% based on geometric means and 99.4% based on arithmetic means (Table 3).

**Table 3:** Efficacy against *Otodectes cynotis* based on geometric mean and arithmetic median number of mites recovered from both Group 1 (negative control) and Group 2 (afoxolaner-treated) dogs

Geometric			Arithmetic		
Group 1 mean	Group 2 mean (% efficacy)	*P*-value	Group 1 mean	Group 2 mean (% efficacy)	*P*-value
22.3	0.3 (98.5)	0.0041^a^	109.9	0.6 (99.4)	0.0084^b^

^a^One-way ANOVA: *F*_(1,14)_ = 11.74, *P* = 0.0041

^b^Non-parametric analysis using the Mann-Whitney test: *W*_(1)_ = 43.5, *Z* = -2.6354, *P* = 0.0084

## Discussion

*Otodectes cynotis* is a commonly occurring surface-dwelling mite that can easily transfer between suitable hosts [1–3]. Since these mites can exist on infected hosts outside the ear canals [1, 2], in order to effectively treat existing infestations, or potentially prevent infestations, effective systemic acaricidal products should be utilized. Properly ascertaining the overall efficacy of antiparasitic products often requires multiple studies be performed. Typically, the first such study performed is a controlled laboratory study utilizing the minimum target dose, realizing that studies of different scales will follow, should the results indicate a potential for success.

This study was designed to assess the efficacy of the minimum dose of afoxolaner against existing infestations of *O. cynotis* in dogs under controlled, laboratory conditions. The present study demonstrates that treatment with a single oral dose (at or approaching the minimum recommended dosage of 2.5 mg/kg) of afoxolaner (NexGard® Chewables) resulted in a rapid reduction in ear mite numbers, suggesting that a single oral administration of NexGard is highly effective (> 98%) against *O. cynotis* infestations in dogs, as assessed 28 days post-administration. This is comparable to efficacy achieved when afoxolaner was tested against other mite species infesting dogs, namely *Sarcoptes scabiei* (100% efficacy based on mite counts) [12] and *Demodex* spp., with > 99% efficacy based on mite counts in a well-controlled study [12] and resolution of cases under field conditions [14].

## Conclusion

The high level of efficacy achieved with a single, minimum dose of NexGard® (afoxolaner) in the present study would seem to justify additional work to gather further evidence of the treatment and control capabilities of afoxolaner against *O. cynotis*, also when considering high efficacy obtained against other mite species infesting dogs.
